# Bioprospecting of *Sechium* spp. varieties for the selection of characters with pharmacological activity

**DOI:** 10.1038/s41598-021-85676-7

**Published:** 2021-03-17

**Authors:** María Isabel Iñiguez-Luna, Jorge Cadena-Iñiguez, Ramón Marcos Soto-Hernández, Francisco Javier Morales-Flores, Moisés Cortes-Cruz, Kazuo N. Watanabe, Ryoko Machida-Hirano, Jorge David Cadena-Zamudio

**Affiliations:** 1 Postgrado de Innovación en Manejo de Recursos Naturales Colegio de Posgraduados, Campus San Luis Potosí, Salinas de Hidalgo, Iturbide 73, 78620 San Luis Potosí, Mexico; 2 Programa de Botánica Colegio de Postgraduados, Km. 36.5 Carretera México-Texcoco, 56230 Texcoco, Estado de México Mexico; 3 Centro Nacional de Recursos Genéticos-INIFAP, Boulevard de la Biodiversidad 300, 47600 Tepatitlán de Morelos, Jalisco Mexico; 4 Grupo Interdisciplinario de Investigación en Sechium edule en México, A.C. Agustín Melgar 10 Col. Niños Héroes, 56108 Texcoco, Estado de México Mexico; 5 Tsukuba Plant Innovation Research Center, University of Tsukuba, Tsukuba, 1-1-1 Tennodai, Tsukuba, Ibaraki Prefecture 305-8571 Japan; 6 Genetic Resources Center, National Agriculture and Food Research Organization, 2-1-2 Kannondai, Tsukuba, Ibaraki 305-8602 Japan; 7 Instituto de Ecología, A. C., Carretera Antigua a Coatepec 351, El Haya, 91070 Xalapa, Veracruz Mexico

**Keywords:** Biochemistry, Cancer

## Abstract

Bioprospecting identifies new sources of compounds with actual or potential economic value that come from biodiversity. An analysis was performed regarding bioprospecting purposes in ten genotypes of *Sechium* spp., through a meta-analysis of 20 information sources considering different variables: five morphological, 19 biochemical, anti-proliferative activity of extracts on five malignant cell lines, and 188 polymorphic bands of amplified fragment length polymorphisms, were used in order to identify the most relevant variables for the design of genetic interbreeding. Significant relationships between morphological and biochemical characters and anti-proliferative activity in cell lines were obtained, with five principal components for principal component analysis (SAS/ETS); variables were identified with a statistical significance (< 0.7 and Pearson values ≥ 0.7), with 80.81% of the accumulation of genetic variation and 110 genetic bands. Thirty-nine (39) variables were recovered using NTSYSpc software where 30 showed a Pearson correlation (> 0.5) and nine variables (< 0.05), Finally, using a cladistics analysis approach highlighted 65 genetic bands, in addition to color of the fruit, presence of thorns, bitter flavor, piriform and oblong shape, and also content of chlorophylls *a* and *b*, presence of cucurbitacins, and the IC_50_ effect of chayote extracts on the four cell lines.

## Introduction

In recent years, evidence of functional biological activity properties has been found in some species of the *Sechium* P. Browne (Cucurbitaceae) genus, whose greatest biodiversity is recorded in Mesoamerica^1–3^; its fruits are called chayote, and their domestication has focused on its use as food. This species has been used in^4^ cardiovascular diseases^5^, antiulcerous^6^, antibacterial^7^ and hepato-protection^8–10^ bio-protection due its antioxidant capacity^11^. The functional properties of chayote mainly in leaves and fruits depend on the content of amino acids, peroxidases, sterols, saponins, phenols, polyphenols, flavonoids^12^ and tetracyclical terpenes^13–18^. These metabolites are of pharmacological interest because of their therapeutic properties, and triterpenes have the highest effect on the cancerous cell lines^19–21^. Currently, there is some interest in developing anti-carcinogenic compounds from natural sources, with the aim of reducing toxic effects or non-selective activities, and resulting in scenarios of pharmaceutical exploitation^21,22^. In the *Sechium* P. Browne genus, ten species are described^3^, from which in Mexico three are present: *S. chinantlense*^23,24^, *S. compositum*^25,26^ and *S. edule*^13,14,27^, with outstanding anti-proliferative activity on carcinogenic lines from fruit extracts. *S. edule*, as a domesticated species has 12 varietal groups due its intra-specific variation: *nigrum levis, albus levis, albus dulcis, nigrum conus, albus minor, nigrum minor, amarus silvestrys, nigrum maxima, nigrum xalapensis, virens levis, nigrum spinosum,* and *albus spinosum*^13^. Riviello-Flores notes the nutraceutical^17^ properties of aqueous extracts obtained from the *virens levis* and *nigrum spinosum* group, several authors note high biological activity of *S, edule, S. chinantlense, S. compositum* and bitter flavored hybrids^18,28–30^ on cell lines derived from malignant tumors HeLa, P-388, L929, WEHI-3, J774^31,32^, and Cadena-Zamudio demonstrated the fragmentation of DNA and induction of apoptosis^33^ with raw extracts obtained from *S. spinosum, S. chinatlense, S. compositum* and the hybrid H38707.

In this regard, the bioprospecting exploitation tries to find new sources of chemical compounds, genes, proteins, and others with existing value or potential economic value that come from biodiversity^31,34,35^, and also in the case of the broad diversity of metabolites and variation of concentrations reported in *Sechium*, the challenges are in identifying the relevant morphological, biochemical and genetic variables for exploitation activity, then increasing the chayote use. This generates a high-impact scenario, as well as areas of opportunity by offering natural alternatives for public health as cancer and diabetes which are singled out as the main causes of death with a high cost^36–38^.

Various studies reveal that these multifactorial pathologies can present a reciprocal influence and are health problems that continue to grow^39,40^ therefore addressing issues of such interest requires an analysis of updated information through a meta-analysis, defined as a process of synthesis of the scientific evidence, concerning issues of clinical, administrative or health technology interest, in order to assist in making objective decisions based on quantitative results^41,42^.

Then new research approaches, such as genetic improvement to potentiate those characters that provide functional effectiveness, such as antineoplastic and selective activity on malignant and normal cells^28,33^. The biological activity of ethanol extracts of chayote fruits on normal and malignant cells has been noted^18,28,29^, been an important source of metabolites, with high productivity (54–136 t ha^−1^)^13,14^, then the possibility to obtain cultivars derived from the bioprospecting study. The challenge of the meta-analysis of *Sechium* is to integrate all the variables evaluated in independent studies with significant anti-proliferative, anti-neoplastic and selective biological activity^12,16,28,43,44^. This meta-analysis methodology is necessary because most of the reports do not show the biological variant of *S. edule* used then reducing the possibility of reproducibility of their results. For this reason, the bioprospective meta-analysis presented here facilitates the identification of the genotype, its character and outstanding character status, specifying the statistical validity and reducing possible contradictions in the literature. The aim of this research is to identify morphological, biochemical, and genetic variables with functional biological activity in ten genotypes of *Sechium* spp. In order to design new varieties through outstanding variables from the bioprospective viewpoint.

## Results and discussion

**Figure 1:**
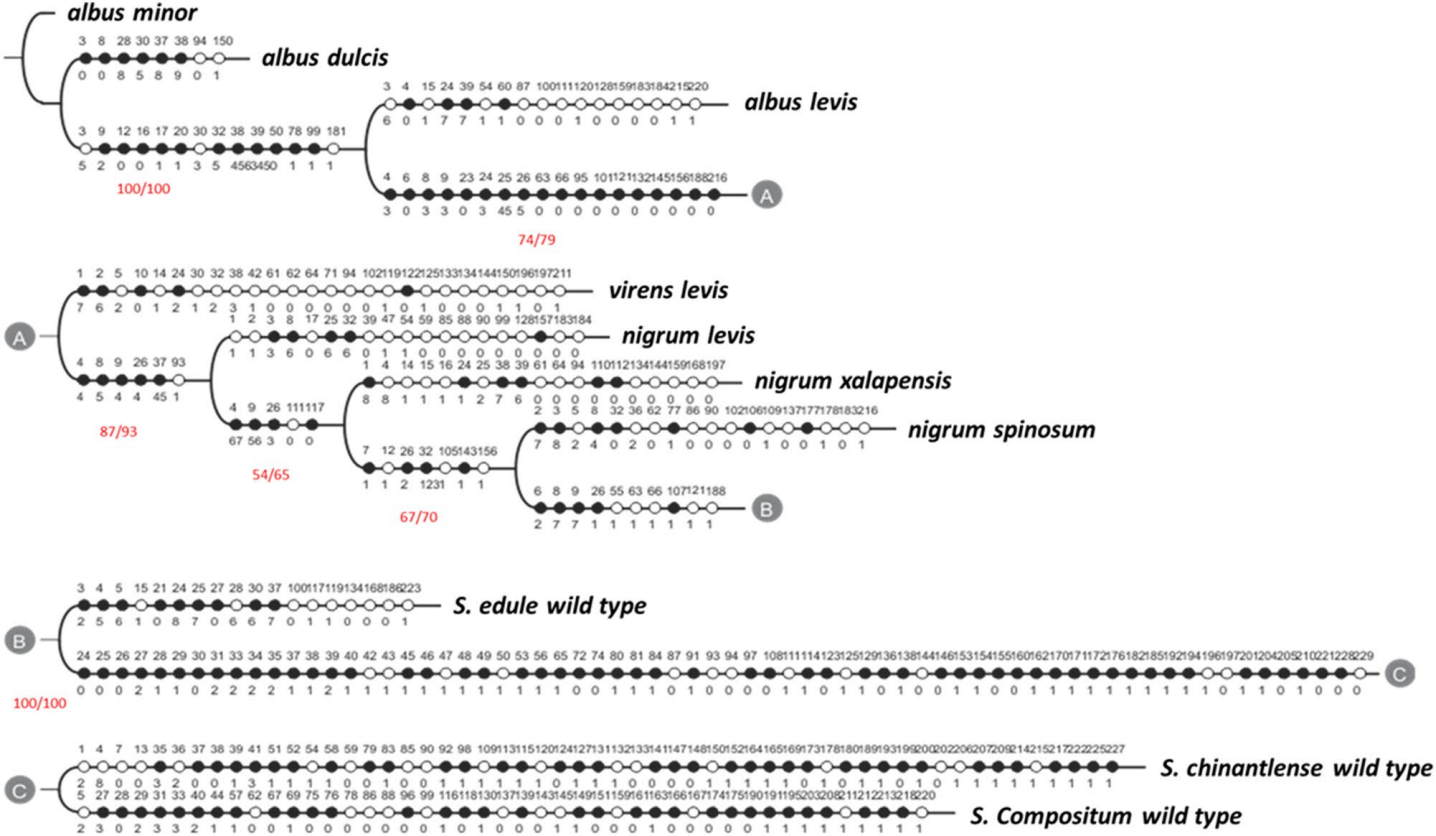
Cladogram of ten genotype of three *Sechium* spp. based on morphological, biochemical, genetic, and biological activity characters on malignant cell lines to identify the character/genotype. White dots represent apomorphic variation and black dots plesiomorphic. The numbers on the higher and lower parts of the cladogram branch represent the character and the state in which it varies. The values separated by the diagonal line represent the Bootstrap/Jackknife indexes, with L = 566, Ci = 59 and Ri = 51.

The distribution of *S. chinantlense, S. compositum and S. edule* for morphological, biochemical, functional biological activity and genetic characterization variables, displaying a monophyletic tree where *albus minor* is placed as the closest to the basal state of the genotypes or taxa (Fig. 1). This differs from what was reported by Cadena-Iñiguez et al.^27^ where the phylogenetic order places *albus minor*, *albus dulcis* and *albus levis* as the genotypes of greatest morphostructural evolution with regards to the wild relative, based on its adaptive specialization to the environment. In this study the fruit of yellow genotypes were those nearest to the root, because they show the lowest number of characters associated to the bioprospecting variables of proliferation, IC_50_, and the optimal concentration (extract doses applied to malignant cell lines with lowest percentage of proliferation). The Bootstrap/Jackknife re-sampling methods showed a high degree of parsimony, with values from 100/100 as maximum to 57/64 as minimum, based on homoplasies, with length L = 566, Consistency Index (CI 59), and Retention Index (RI 51)^45^ for apparently similar characters that result from independent evolution. The three genotypes from the *albus* group reflected a lower biological activity per lower number of characters associated (Table 1), except *albus levis* which showed a content of cucurbitacin P (CbP) as an autopomorphic character.

**Table 1:** Apomorphic characters from the heuristic clade with morphological, biochemical, and genetic variables in ten genotypes of *Sechium* spp.

Genotype	Character/character state	Morphological	Biochemical	AFLPs bands	Functional biological activity
*S. edule albus minor*	43/1, 93/1, 105/1, 178/1			43, 93, 105, 178	
*S. edule albus dulcis*	2/3, 150/1, 187/1, 206/1, 229/1	Width 5.65 cm		150, 187, 206, 229	
*S. edule albus levis*	2/3, 15/1, 30/3, 37/2, 54/1, 120/1, 181/1, 187/1, 206/1, 215/1, 220/1	Width 5.65 cm	CbP	54, 120, 181, 206, 215, 220	P-388 proliferation 34% HeLaIC_50_ 500 µg mL^−1^
*Sechium edule*	6/2, 7/1, 8/7, 9/7, 13/1, 15/1, 105/1, 107/1, 119/1, 126/1, 143/1, 198/1, 224/1	Bitter, spine, width 9.0 cm	Chlorophyll a 0.223 mgg^−1^, Chlorophyll b 0.2458 mgg^−1^ DHCbE,CbP, Color198°Hue	105, 107, 119, 126, 143, 198, 224	
*S. edule nigrum levis*	47/1, 54/1			47, 54	
*S. edule virens levis*	5/2, 37/2, 42/1, 119/1, 125/1, 150/196/1, 1, 211/1	Piriform		42, 119, 125, 211	HeLaIC_50_ 500 µg mL^−1^
*S. edule nigrum xalapensis*	4/8, 5/4, 15/1, 16/1	Oblong	Color 223°Hue, CbP, ICbE		
*S. edule nigrum spinosum*	5/2, 7/1, 18/1, 36/2, 109/1, 143/1, 216/1	Piriform, thorns	ICbD	94, 144, 197, 216	Proliferation WEHI-3 20%
S. *chinantlense*	1/2, 2/2, 4/8, 5/4, 54/1, 70/1, 109/1, 120/1, 126/1, 132/1, 142/1, 143/1, 150/1, 178/1, 198/1, 206/1, 215/1, 224/1	Length 5.94 cm, width 5.2 cm, Oblong	Color 223°Hue, Chlorophyll a 0.119 mg g^−1^	54, 70, 86, 109, 120, 126, 132, 142, 143, 150, 178, 206, 215, 224	
S. *compositum*	5/2, 7/1, 90/1, 145/1, 211/1, 220/1	Piriforme, spine		90, 145, 211, 220	

The diagonal indicates the character and the relevant state of the character.

Starting from the fourth clade (*nigrum levis, S. edule, virens levis, nigrum xalapesis*), a greater association to the percentage of proliferation and IC_50_ on cell lines and characters was observed, related to the piriform shape of the fruit, presence of cucurbitacins I (CbI), cucurbitacins D (CbD), dihydrocurcubitacin E (DHCbE), high content of chlorophylls *a* and *b*, soluble solids and total carotenoids, of synapomorphic origin^45–47^, which could evidence effects from their human manipulation, creating different life histories in the genotypes, fostering the variation of *S. edule* as an intraspecific complex (Tables 1 and 2). The ancestral characters of symplesiomorphic origin, such as the dark green color, total solids, ascorbic acid, chlorophylls *a* and *b*, and titratable acidity, are related to the percentage of proliferation, IC_50_, cell lines HeLa, P388, L929, highlighting that for *S. chinantlense* and *S. compositum* the characters of piriform shape of the fruit, polymorphic bands and percentage of proliferation and IC_50_, stand out in their association with the leukemia line WEHI-3, HeLa, J774 and L929 (Tables 3 and 4). *S. chinantlense* and *S. compositum* showed relevant morphological and biochemical characters in the clades, in contrast with *S. edule* and their varietal groups, possibly because the first two are species without human manipulation, and because, to the best of our knowledge, there is no reported evidence of their use as food or medicine, and they have not developed morphological variation as in the case of *S. edule*^48^. Mendoza et al. and Soler^49^ note that the morphological and biochemical characters result from the genetic expression and regarding this, the same clade shows that the polymorphic bands (between 42 and 227) for *S. chinantlense* and those (between 44 and 218) for *S. compositum* are ancestral states of speciation^49^. The characters located in the branches prior to the formation of a clade^47,50^, show that chlorophyll a, b, cucurbitacins E (CbE), cucurbitacins B (CbB), dihydrocurcubitacin D (DHCbD), dihydrocurcubitacin B (DHCbB), total solids, ascorbic acid and dark green to very dark green color and bitter flavor^3,13^ are associated to variables of therapeutic interest^32,51^. The apomorphic characters are biological traits that are evolutionarily novel and are derived from the phylogenetically most proximal ancestral taxon^46,47^. The apomorphic characters (Table 4) present few characters and states of character associated to variables of functional biological activity, showing five morphological (thorns, bitter, piriform, width and length of the fruit), eight biochemical (higher concentrations of chlorophyll a and b, cucurbitacin P (CbP), isocucurbitacin E (ICbE), isocucurbitacin D (ICbD), DHCbE, light green color and dark green color of the fruit), and biological activity variables (such as percentages of proliferation in lines P-388, WEHI-3, and IC_50_ in HeLa); meanwhile, the symplesiomorphic characters (Table 2) that show a trait shared by two related taxa, when it coincides with the character present in the common ancestors of both, showed a higher number within the cladogram, revealing an association with the antiproliferative effect on P-388, L-929, J-774, HeLa and WEHI-3, IC_50_, and more efficient dose, with different character states per genotype, such as the oblong shape and large size of the fruit, while those of biochemical origin showed diverse values of chlorophyll a and b, with influence on the variation of the fruit color from yellow to green with different intensities, ascorbic acid, total solids, titratable acidity, presence of CbB, DHCbB and DHCbD. Then the ancestral characters showed a higher variability identified by the cladistics approach as bioprospecting variables that determine their functional biological activity.

**Table 2:** Apomorphic and plesiomorphic characters identified in the branches of the heuristic clade based on morphological, biochemical, and genetic variables of ten *Sechium* spp. genotypes.

Branch	Character apomorphic	Branch	Character plesiomophic
1	Length 5.77 cm, Wide 5.2 cm, 55,70,206	1	Chlorophyll b 0.00418 mg g^−1^, CbE; 71,134,202,223, HeLa proliferation 21%
2	n/a	2	Depth 3.40 cm; Chlorophyll b 0.00541 mg g^−1^, DHbD, CbB, Total solids 8.08°Brix, 78,99,104; L929 proliferación 44%, P388 IC_50_ 1010 µg mL^−1^, L929 IC_50_ 370 µg mL^−1^
3	93,229	3	Color 167.2°Hue, Chlorophyll a 0.119 mg g^−1^, Chlorophyll b 0.0846 mg g^−1^, DHCbB, Titratable acidity 0.038%, %, ascorbic acid 6.53 mg g^−1^; L929 proliferation 26%, HeLa IC_50_ 840 µg mL^−1^
4	P-388 Proliferation 15%, 100, 111	4	Depth 4.20 cm, Neutral, total solids 5.47°Brix, HeLa proliferation 15%
5	198.4°Hue, DHCbE, 128	5	Length 8.1, wide 6.8 cm, depth 5.34, 186, P388 proliferation 18%, L929 proliferation 22%
6	Thorns, ICbD, 187, 198	6	Length 13.55 cm, wide 8.19, CbQI, total solids 5.14°Brix; L929 IC_50_ 1400 µg mL^−1^
7	Bitter, 101, 105	7	Color 200°Hue, Chlorophyll b 0.0922 mg g^−1^, Ascorbic acid 4.95 mg g^−1^,135,226¸L929 proliferation 18%, J774 proliferation 10%, P388 IC_50_ 480 µg mL^−1^, WEHI-3 IC_50_ 0.93 µg mL^−1^
8	Chlorophyll a 0.223, Chlorophyll b 0.2458, 42, 43, 47, 50, 55, 63, 66, 107, 121, 125, 187, 188, 196	8	45, 46, 48, 49, 56, 65, 80, 81, 84, 91, 114, 129, 138, 153, 154, 162, 170, 171, 172, 176, 182, 185 ,192, 194, 201, 204, 210, 221, 228 HeLa [2400 µg mL^−1^], HeLa proliferation 6%, P388 [2400 µg mL^−1^], L929 [2.5 µg mL^−1^], J774 [5.0 µg mL^−1^], J774 proliferation 17%, WEHI-3 [2.5 µg mL^−1^], HeLa IC_50_ 1.51 µg mL^−1^, P388 IC_50_ 0.98 µg mL^−1^, L929 IC_50_ 6.37 µg mL^−1^, J774 IC_50_ 0.26 µg mL^−1^

The diagonal indicates the character and its relevant character state.

**Table 3:** Symplesiomorphic characters from the heuristic clade generated from morphological, biochemical, and genetic variables of ten *Sechium* spp. genotypes.

Genotype	Character/status	Morphological (cm)	Biochemical	AFLPs bands	Functional biological activity
*S. edule albus dulcis*	1/5, 3/0, 8/0, 24/5, 28/8, 30/5, 37/8, 38/9	Length 8.28	Chlorophyll a 0.00234 mg g^−1^		P388 proliferation 45% HeLa IC_50_ 1510 µg mL^−1^, P-388 IC_50_1980 µg mL^−1^
*S. edule albus levis*	3/6, 4/0, 24/7, 26/7, 28/7, 38/4, 39/7, 60/1	Depth 5.50	Color 60.8°Hue, Total solids 8.08°Brix, Ascorbic acid 7.82 mg g^−1^	60	HeLa proliferation 17%, P388 IC_50_ 940 µg mL^−1^, L929 IC_50_ 1900 µg mL^−1^
*Sechium edule*	1/3, 4/5, 5/6, 24/8, 25/7, 30/6, 32/3, 37/7, 39/4	Length 7.8 Ovoid	Color 198.4°Hue, Total solids 10.92°Brix, Titratable acidity 0.059%		P388 proliferation 47%, L929 proliferation 84%, HeLa IC_50_ 1170 µg mL^−1^, L929 IC_50_ 1100 µg mL^−1^
*S. edule nigrum levis*	1/1, 2/1, 8/6, 25/6, 26/4, 32/6, 37/5, 38/6,	Length 5.77 Width 4.47	Chlorophyll a 0.198 mg g^−1^, Titratable acidity 0.045%, Ascorbic acid 6.65 mg g^−1^		L929 proliferation 51%, HeLa IC_50_ 880 µg mL^−1^, %, P388 IC_50_ 880 µg mL^−1^
*S. edule virens levis*	2/6, 4/3, 8/3, 9/3, 25/5, 26/5, 28/4, 38/3	Width 8.74	Color 126.5°Hue, Chlorophyll a 0.06 mg g^−1^, Chlorophyll b 0.0712 mg g^−1^, Titratable acidity 0.04%,		HeLa proliferation 17%, P388 IC_50_ 730 µg mL^−1^
*S. edule nigrum xalapensis*	1/8, 3/7, 9/6, 24/1, 25/2, 30/2, 32/4, 38/7, 39/6	Length14.96, depth 5.85	Chlorophyll b 0.1231 mg g^−1^, Total solids 4.93°Brix, Titratable acidity 0.032%,		P388 proliferation 27%, L929 proliferation 34%, P388 IC_50_ 1120 µg mL^−1^, L929 IC_50_ 1800 µg mL^−1^
*S. edule nigrum spinosum*	1/6, 2/7, 3/8, 4/7, 8/4, 24/4, 28/5, 77/1	Length 10.54, Width 9.00, Depth 6.24,	Total solids 6.43°Brix,	77	HeLa proliferation 19%,
*S. chinantlense*	3/4, 35/3, 41/3, 51/1, 52/1, 58/1, 83/1, 92/1, 98/1, 113/1, 115/1	Depth 4.6		51, 52, 58, 83, 9, 98, 113, 115, 127, 131, 152, 164, 165, 169, 189, 200, 207, 209, 217, 222, 225, 227	WEHI-3 0.3 µg mL^−1^
*S. compositum*	27/3, 28/0, 29/2, 31/3, 33/3, 40/2, 44/1, 57/1, 69/1, 116/1, 130/1, 149/1, 163/1			44, 57, 69, 116, 130, 149, 163, 191, 195, 203, 208, 212, 213, 218	HeLa 5.0 µg mL^−1^, HeLa proliferation 6%, L929 10.0 µg mL^−1^, J774 10.0 µg mL^−1^, J774 IC_50_ 1.96 µg mL^−1^

**Table 4:** Characteristic values and their accumulated proportion for five first principal components from the analysis of 229 morphological, biochemical, genetic, and biological activity variables of ten *Sechium* spp. genotypes.

PC	Eigenvalues	Difference	Variance	Cumulative variance	%
1	48.7115	12.0101	0.2849	0.2849	28.49
2	36.7014	12.6146	0.2146	0.4995	49.95
3	24.0868	8.1462	0.1409	0.6403	64.03
4	15.9406	3.2017	0.0932	0.7336	73.36
5	12.7389		0.0745	0.8081	80.81

Meta-analyses are a tool used in the area of health and natural products to synthesize published information regarding medication and adverse drug reactions^52,53^. They have recently been applied to the agricultural sector to facilitate the nutritional improvement of crops (chickpea and avocado) and have outlined varied responses to factors analyzed from independent studies, such as micronutrients and mycorrhizal symbiosis^54–57^. Other studies try to elucidate new agri-food applications such as soil amendment, water use, crop selection and natural resource optimization over time and across different regions^58,59^.

In this manner, our results offer an approach with greater statistical parsimony and relevance to identify outstanding variables *in Sechium* spp., based on accurate information, with traceable data and reproducible results, avoiding bias to acquire a scientifically valid view^57,60^.

### Multivariate analysis

The multivariate analysis allowed identifying the variables that explain the higher total variability contained in the data and exploring the correlations and reducing the dimension of the analysis with new indexes. It was determined that with five principal components (PCs), the accumulated value of 80.81% of the variation was obtained (Tables 4, 5).

**Table 5:** Characteristic vectors of the analysis of 229 morphological, biochemical, genetic, and biological activity variables of fruits from ten *Sechium* spp. genotypes.

Character	PC1	PC2	PC3	PC4	PC5
Fruit length	− 0.04524	− 0.00769	− **0.16111**	− 0.06994	0.02867
Fruit width	− 0.03345	0.01857	− **0.16263**	0.01423	0.02682
Fruit depth	0.00121	0.03002	− **0.13941**	0.03266	0.05199
Fruit color	0.05023	0.04151	− **0.14366**	0.05107	0.07284
Fruit shape	0.03729	− 0.05812	0.03033	− 0.01781	**0.18951**
Fruit flavor	0.07670	0.07707	0.09244	0.00519	**0.10792**
Thorns presence	− 0.02267	0.08481	− 0.04393	**0.15985**	0.09606
Chlorophyll b	**0.07228**	0.07928	− 0.07577	0.02160	**0.12088**
DHCbE	− 0.05068	0.05222	− **0.14751**	0.01722	0.07887
Cucurbitacin QI (CbQI)	− 0.03738	− 0.03189	− **0.13406**	− **0.16161**	− 0.01147
DHCbD	0.02997	0.03844	− **0.12153**	0.00719	**0.15736**
ICbD	− 0.04531	− 0.03732	− **0.16878**	− 0.00500	− 0.04508
DHCbB	0.05486	0.05835	− 0.05079	**0.15351**	0.05338
CbB	0.02221	0.02730	− **0.14062**	0.02372	**0.14749**
Curcubitacin L (CbL)	0.00793	0.02068	− 0.01228	− 0.04253	− **0.19613**
CbE	0.03684	0.03955	0.00237	0.06428	**0.15947**
Total carotenoids	− 0.02551	− 0.03265	**0.16134**	− 0.02069	− **0.10832**
More efficient dose for HeLa	− **0.09732**	− **0.10710**	0.00724	− 0.00483	− 0.06686
Proliferation for P388	− 0.05457	− 0.04417	**0.14698**	− 0.00224	0.07366
Proliferation for L929	0.06711	0.02431	**0.14143**	− 0.06525	− **0.11086**
More efficient dose for J-774	− 0.01927	− 0.01384	− 0.07914	**0.20791**	− 0.05360
More efficient dose for WEHI-3	− 0.01909	− 0.01434	− 0.07914	**0.20788**	− 0.05361
Proliferation for WEHI-3	**0.08871**	0.04309	− 0.07063	**0.14569**	− 0.04878
IC50 for L929	− 0.04620	− 0.05157	− 0.06058	− 0.09235	**0.16805**
IC50 for WEHI-3	− 0.01933	− 0.01430	− 0.07910	**0.20785**	− 0.05358

The values in bold are those that were statistically the most significant because of their principal component.

**Figure 2:**
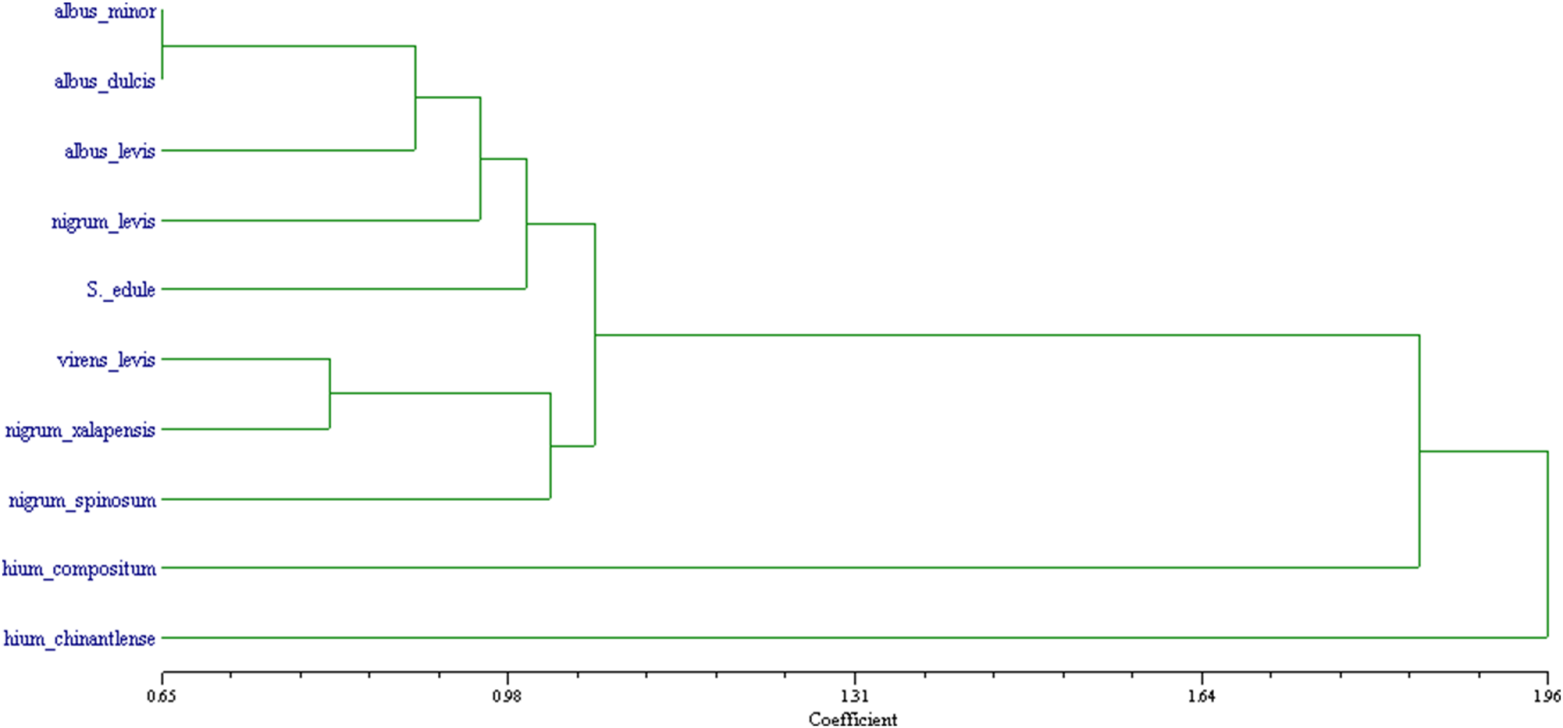
Dendrogram from the coefficient of association or similarity, with the NTSYSpc^62^ software, based on 229 multi-state morphological, biochemical, genetic, and biological activity variables of fruits from ten *Sechium* spp. genotypes.

The five PCs identified 110 polymorphic bands with higher statistical weight (not showed); however, in an environment of parent selection for genetic improvement, it is relevant to identify the bands associated in each component with bioprospecting variables (Table 5). The grouping methods seek the formation of groups of basic characterization units (BUCs) with characteristics to similarities or differences between pairs, whether through the matrix of indexes^61^. In this regard, the dendrogram (Fig. 2) shows four groups of genotypes, showing that *S. chinantlense* and *S. compositum* are separate due to their wild origin with regards to the *S. edule* genotypes, and showing a greater distance, equivalent to a lower similarity index. Although they share morphological and biochemical characters (color, bitter flavor, cucurbitacins) with *S. edule*, the latter was found to be far placed, sharing a higher number of characters with its domesticated variants. The edible chayote *n. xalapensis*, *v. levis* and *n. spinosum* stand out with green fruits, larger size and larger thorns in the latter, which are related with greater proximity with the two wild species because of their biological activity^16,24^, while the yellow fruit genotypes are those with the most dissimilarity and the least relevance for bioprospecting variables with 10–100% less content of cucurbitacins than green chayote variants and their wild ancestor (*S. edule*), respectively^15^, hence for the purpose of anti-proliferative activity they are not considered as bioprospectively relevant.

In Figs. 1 and 2 show a similar distribution for the *Sechium* spp. genotypes, where only the position of *S. chinantlense* was inverted with *S. compositum*; however, they do not change branch in the grouping, thus conserving the distances, and this also occurs in *S. edule* and *nigrum levis.* Compared to the multivariate analysis, the cladistics approach shows the relevant character and its state, which improves the identification of the variable associated to the functional biological activity (bioprospecting variables), which can help to discriminate or decide its importance in programs of basic research or genetic improvement. The cladistics analysis helps to identify phylogenetic relationships with the evolutionary approach of taxa^47^; however, in this case, the cladogram identifies the possible bioprospecting characters, which strongly influence the relationship between taxa, showing that the wild genotypes present characters of higher statistical weight suggested as bioprospecting variables in comparison to *S. edule* and its domesticated genotypes, from whose group some stand out solely because of their higher biological efficiency, *n. xalapensis, virens levis and n. spinosum*, which are identified as the edible types with the highest level of domestication; meanwhile, *a. minor, a. dulcis and a. levis,* whose morphological, biochemical and genetic characters do not contribute significantly to the bioprospecting variables, are more related to the environmental effect^27^ (Fig. 2). This also highlights that they are more distant from *S. chinantlense* and *S. compositum* of bitter flavor, as well as from the groups of *S. edule* with green fruits, where the wild genotypes synthesize a higher amount of secondary metabolites^63–65^ in comparison to the domesticated variants^66^. Among the terpenes there is a group of triterpenes known as cucurbitacins, closely related to the Cucurbitaceae family, and the bitter flavor of the wild types of *Sechium* is attributed to these.

The important bioprospecting variables in the selection of parents for genetic crossing: fruits that are large, piriform, oblong of dark green color, with thorns and bitter flavor, or else those that present the highest levels of chlorophyll a and b, presence of cucurbitacins (ICbE, |1,234,567,890′sa, CbE, ICbD, CbB, DHCbB, DHCbD and CbQI), assuming a possible association with the corresponding polymorphic bands^63^. The analyses that determine distances have received criticism because they do not take into account the evolutionary processes, since several characters that are correlated do not evolve independently^67^, while the cladistics methods tend to analyze all the characters individually, contrary to the reduction of information to be explained through indexes of genetic distances^68^. It is important to highlight that many times the same pattern can be explained through different population histories^46^, which suggests delving into aspects that have a relationship with the number of genes that determine a character. This suggests a continuous dynamic interaction of adaptation with the factors in which the population grows, and each species adjusts the information contained in the genome according to its needs for survival; therefore, the management of descriptors, characteristics or measurable attributes is important, in order to record and evaluate the references of shape, structure or behavior of a genotype, because these are of interest for plant breeders and agronomists^69^. Our results suggest that even when the genotypes are quite near taxonomically, particularly in the *S. edule* complex, there are notable differences in their biochemical components, evidencing a diversity of metabolites that can be used in applications other than food, for example for therapeutic or pharmacological uses.

In a pharmacological study, Lira^2^ revealed the diuretic properties of the seeds, as well as cardiovascular and anti-inflammatory of leaves and fruits. Also been observed that extracts from *S. edule* have activity against gram-positive bacteria^70^, while during a *vitro* study^11^ proved their antioxidant activity. Other studies carried out on Wistar rats, demonstrated that *S. edule* extracts have hypotensive activity^71^, in addition to the ability to alter the marking of blood elements with the technetium-99 radionuclide, to modify the morphology of erythrocytes, promote the fixation of radioactivity in blood proteins and the biodistribution of pharmaceutic radio sodium pertechnetate. Other studies have shown that these extracts induce damage to the DNA molecule^28,33^, reduce the levels of glucose, globulin, and diastolic blood pressure.

In sum, it is suggested that the biological effects shown can be because chayote presents in its composition compounds that act as active metabolites in vivo with antioxidant properties^72–76^. Phytochemical studies in *S. edule* have identified non-phenolic alkaloids, saponins, sterols, triterpenes^4^ and eight glycosylate flavonoids^12^, whose pattern of compounds is shared by taxonomically related species^62^ and many of them are associated to anti-tumor activity^77^. Studies allowed isolating and characterizing, from the chayote seed, the sechiumine protein, a molecule with the property of deactivating the ribosomal function in the cervical cancer line HeLa, and it was located as a possible chemotherapeutic agent^78^. This is quite relevant and contributes widely to the search for alternatives from natural sources for the treatment of public interest illnesses; however, in 99% of the pharmacological and phytochemical studies that reported for *S. edule*, the biological type or varietal group evaluated are not described, presenting difficulties for experiment reproducibility, and risks regarding its pharmacological application in humans. It is relevant to note the importance of a taxonomic discipline to classify the genotypes of an intraspecific complex, as is the case of *S. edule,* since it facilitates its identification through secondary characters.

## Conclusions

The meta-analysis carried out, identified as principal bioprospecting variables the dark green color, bitter flavor, presence of thorns, oblong and piriform shape of the fruits. Also, the presence of eight cucurbitacins (ICbE, DHCbE, CbE, ICbD, CbB, DHCbB, DHCbD and CbQI), and the highest values of chlorophyll a, and b. The results from polymorphic bands showed 49 for the apomorphic characters and 38 for the symplesiomorphic in the genotypes evaluated. It is very important to consider the taxonomic identification (inter and intraspecific) of biological variants, mainly for *S. edule* genotypes, since they present large morphobiochemical, genetic and functional biological activity differences, which can be used for various investigations.

## Experimental section

### General procedures

The meta-analysis contributes to a systematic, objective, and scientific method for the quantitative revision of primary studies with a common theme^79,80^, considering a rigorous synthesis of the best possible evidence^81^. For this analysis, the information concerning *Sechium* was catalogued under the initial criterion of identification of the species and genotype employed (varietal). The search was performed in the databases of Google Scholar, CAB Abstracts, Agris, Web of Science, Biological Abstracts, Microsoft Academy, and Scopus with the keywords *S. edule*, *S. edule* secondary metabolism*, S. chinantlense, S. compositum*, chayote, varieties, apoptosis, anti-proliferative, characterization, cucurbitacins, DNA extraction, peroxidases, phenols, flavonoids, and chromatography. From these, 7427 results were identified, and when applying the criteria of species and varietal group identified in each publication, the sample was reduced to 20 (Table 6). With this, a database made up of five morphological variables of fruits (flavor, color, shape, size and thorns) was elaborated, as well as 19 biochemical variables (chlorophyll a, b, presence of 13 cucurbitacins, total carotenoids, total solids, titratable acidity and ascorbic acid), three determinations for the biological evaluation of plant extracts (percentage of proliferation, IC_50_ and dose of highest anti-proliferative effect) on malignant cervical uterine cell lines (HeLa), leukemia in mice (P388), fibroblasts (L929), sarcoma (J774) and leukemia (WEHI-3), in addition to normal mice bone marrow cells, and 188 bands of polymorphic genetic characterization through AFLPs for *S. chinantlense, S. compositum* and eight varieties of *S. edule* (*albus minor, albus dulcis, albus levis, nigrum levis, nigrum xalapensis, nigrum spinosum, virens levis and S. edule wild type*). The concentration units reported from the different reports were standardized to µg mL^−1^ (Table 7).

**Table 6:** Publications included for the bioprospecting meta-analysis in *Sechium* spp.

Variable	Genotype	Research carried out	References
Biological activity	*Sechium edule* (*albus minor; albus dulcis; albus levis; virens levis; nigrum levis; nigrum xalapa; nigrum spinosum; S. edule*); *S.compositum; S.chinantlense*	Bioassay in cancerous cell lines P388; J774 WEHI-3; HeLa; P388; L929; MCF7 Evaluation of apoptosis and determination of DNA fragmentation	^17,19,24–31,33,34,48–50^
Biochemical	*Sechium edule* (*albus minor; albus dulcis; albus levis; virens levis; nigrum levis; nigrum xalapa; nigrum spinosum;S. edule*); *S.compositum; S.chinantlense*	Determination of biochemical profile (cucurbitacins, Chlorophyll b and a, carotenoids, total solids, titratable acidity, ascorbic acid)	^14–19,24,25,30^
Morphological	*Sechium edule* (*albus minor; albus dulcis; albus levis; virens levis; nigrum levis; nigrum xalapa; nigrum spinosum;amarus silvestrys*); *S.compositum; S.chinantlense*	Description of fruit characters based on descriptors (UPOV)	^14,28^
AFLPs	*Sechium edule* (*albus minor; albus dulcis; albus levis; virens levis; nigrum levis; nigrum xalapa; nigrum spinosum;S. edule*); *S.compositum; S.chinantlense*	Determination of 228 AFLP bands	^14^

*UPOV* International union for the protection of new varieties of plants, *AFLP* Amplified fragment length polymorphism.

**Table 7:** Morphological, biochemical, biological effectiveness and genetic characterization variables in *S. edule*, *S. chinantlense*, *S. compositum* for the bioprospecting analysis.

No	Character	Character state	No	Character	Character state
1	Fruit length (cm)	0 = 3.07;1 = 5.77;2 = 5.94; 3 = 5.99; 4 = 7.8;5 = 8.1; 6 = 8.28; 7 = 10.54; 8 = 13.55; 9 = 14.96	2	Fruit width (cm)	0 = 2.70;1 = 4.47;2 = 5.2; 3 = 5.4;4 = 5.65; 5 = 5.82; 6 = 6.8; 7 = 8.19; 8 = 8.47;9 = 9.0
3	Fruit depth (cm)	0 = 0; 1 = 3.12; 2 = 3.40; 3 = 4.20; 4 = 4.60; 5 = 5.30; 6 = 5.34;7 = 5.50; 8 = 5.85; 9 = 6.24	4	Color (°Hue)	0 = 60.8; 1 = 67.3; 2 = 68.3;3 = 126.5; 4 = 167.2;5 = 198.4;6 = 200;7 = 201.1;8 = 222.9;9 = 223
5	Fruit shape (UPOV)	2 = Pirifom; 3 = Spherical; = Oblong; 6 = ovoid	6	Fruit flavor (UPOV)	0 = Neutral; 1 = Sweet; 2 = Bitter
7	Thorns (UPOV)	0 = Absence; 1 = Presence	8	Chlorophyll a (mg g^−1^)	0 = 0.00234; 1 = 0.00362; 2 = 0.0175; 3 = 0.06; 4 = 0.084; 5 = 0.119; 6 = 0.198; 7 = 0.223
9	Chlorophyll b (mg g^−1^)	0 = 0.0034;1 = 0.00418; 2 = 0.00541;3 = 0.0712; 4 = 0.0846; 5 = 0.0922; 6 = 0.1231;7 = 0.2458	10	Dihidrocucurbitacin I (DHCbI)	(0 = Absence;1 = Presence)
11	Glicósidocucurbitacin B (GCbB)	(0 = Absence;1 = Presence)	12	GlicoCucurbitacin E GLCbE)	(0 = Absence;1 = Presence)
13	Dihidrocucurbitacin E (DHCbE)	(0 = Absence;1 = Presence)	14	Cucurbitacin QI (CbQI)	(0 = Absence;1 = Presence)
15	Cucurbitacin P (CbP)	(0 = Absence;1 = Presence)	16	Isocucurbitacin E (ICbE)	(0 = Absence;1 = Presence)
17	Dihidrocucurbitacin D (DHCbD)	(0 = Absence;1 = Presence)	18	Isocucurbitacin D (ICbD)	(0 = Absence;1 = Presence)
19	Dihidrocucurbitacin B (DHCbB)	(0 = Absence;1 = Presence)	20	Cucurbitacin B (CbB)	(0 = Absence;1 = Presence)
21	Curcubitacin L (CbL)	(0 = Absence;1 = Presence)	22	Curcubitacin E (CbE)	(0 = Absence;1 = Presence)
23	Total carotenoids (mg g^−1^)	0 = Undetermined; 1 = 0.0042;2 = 0.0056; 3 = 0.01028	24	Total solids (°Brix)	0 = Undetermined; 1 = 4.93;2 = 5.14; 3 = 5.47;4 = 6.43; 5 = 7.21;6 = 7.66; 7 = 8.08;8 = 10.92
25	Titratable acidity (%)	0 = Undetermined; 1 = 0.029;2 = 0.032; 3 = 0.035;4 = 0.038; 5 = 0.04;6 = 0.045; 7 = 0.059	26	Ascorbic acid (mg g^−1^)	0 = Undetermined; 1 = 3.99; 2 = 4.95; 3 = 6.53; 4 = 6.65; 5 = 6.76; 6 = 7.42; 7 = 7.75; 8 = 7.82
27	HeLa (μg mL^−1^)	0 = 1180; 1 = 2400; 2 = 2.50; 3 = 5.0	28	HeLa-proliferation (%)	0 = 6; 1 = 10; 2 = 13; 3 = 15; 4 = 17; 5 = 19; 6 = 21; 7 = 22; 8 = 23
29	P-388 (μg mL^−1^)	0 = 2400; 1 = 5.0; 2 = 10.0	30	P388-proliferation (%)	0 = 18; 1 = 21; 2 = 27; 3 = 34; 4 = 40; 5 = 45; 6 = 47
31	L-929 (μg mL^−1^)	1 = 2400; 2 = 2.50; 3 = 10.0	32	L929-proliferation (%)	0 = 11; 1 = 18; 2 = 22; 3 = 26; 4 = 34; 5 = 44; 6 = 51; 7 = 84
33	J-774 (μg mL^−1^)	0 = Undetermined; 1 = 2500; 2 = 5.0; 3 = 10.0	34	J774-proliferation (%)	0 = Undetermined; 1 = 2; 2 = 17
35	WEHI3 (μg mL^−1^)	0 = Undetermined 1 = 2370; 2 = 2.50; 3 = 5.0	36	WEHI3-proliferation (%)	0 = Undetermined; 1 = 10; 2 = 20
37	HeLa IC_50_ (μg mL^−1^)	0 = 0.74;1 = 1.51;2 = 500; 3 = 600;4 = 840;5 = 880; 6 = 930;7 = 1170;8 = 1510	38	P388 IC_50_ (μg mL^−1^)	0 = 0.98; 1 = 2.75; 2 = 480;3 = 730;4 = 940; 5 = 1010;6 = 1060; 7 = 1120;8 = 1970; 9 = 1980
39	L929 IC_50_ (μg mL^−1^)	0 = Undetermined; 1 = 3.33;2 = 6.37;3 = 370; 4 = 1100;5 = 1400;6 = 1800; 7 = 1900	40	J774 IC_50_ (μg mL^−1^)	0 = Undetermined; 1 = 0.26;2 = 1.96
41	WEHI-3 IC_50_ (μg mL^−1^)	0 = Undetermined; 1 = 803.1;2 = 0.93;3 = 0.3	42–229	AFLPs (bands)	0 = Absence 1 = Presence

### Selection criteria

The systematic reviews involved in the meta-analysis were directed toward the use of information already disclosed, to be reanalyzed with new approaches and perspectives for research^58,59^. An important aspect in selecting criteria was that the studies required complete information, with traceable data and reproducible results, to reduce or avoid biases in research^57,60^.

The publications that address the fruits of *S. edule, S. chinantlense* and *S. compositum* as a central theme were considered, excluding studies that did not specify the genotype used, which shared evaluation studies about biological activity with very similar methods, and which considered variables that have allowed some type of varietal classification related to the subject in previous reports (Table 6).

The analysis was developed with two approaches. The first was through a cladistics analysis because it incorporates the Popper critical rationalism approach through the refutation of phylogenic hypotheses examined under a principle of parsimony^82,83^; and through non-parametric statistics and using the WinClada version 1.00.08^84,85^ (free license) software with the Bootstrap/Jackknife re-sampling methods, addressing the genotypes as a population, through a random simulation^86^, performing a random elimination of variables until generating a parsimonious cladogram^67^, to define the stability of the clades and identify the status of the outstanding character(s). The analysis was repeated 1000 times creating a percentage that was used as an index of support, consistency, or confidence in the cladograms^87^.

The second analysis was multivariate with the NTSYSpc version 2.20N^88^ program to identify variables with significance < 0.5 and Pearson values ≥ 0.5, genetic distances through unweighted pair group method with arithmetic mean (UPGMA), and principal components (PC) using SAS/ETS (SAS Institute, Inc., 2008)^89^ in order to identify variables with significance < 0.7 and Pearson values ≥ 0.7.

## Abbreviations

AFLP: Amplified fragment length polymorphism
NTSYS: Numerical taxonomy and multivariate analysis system
HeLa: Malignant cervical uterine cell lines
P388: Leukemia in mice
L929: Mouse fibroblast cell line
J774: Mouse macrophage cell line
WEHI-3: Leukemic cells
IC_50_: Inhibitory concentration, 50%
DNA: Deoxyribonucleic acid
UPOV: International union for the protection of new varieties of plants,
μg mL^−^^1^: Concentration in μg mL^−^^1^
DHCbI: Dihidrocucurbitacin I
GCbB: Glycoside of cucurbitacin B
GLCbE: GlicoCucurbitacin E
DHCbE: Dihidrocucurbitacin
CbQI: Cucurbitacin QI
CbP: Cucurbitacin P
ICbE: Isocucurbitacin E
DHCbD: Dihidrocucurbitacin D
ICbD: Isocucurbitacin D
DHCbB: Dihidrocucurbitacin B
CbB: Cucurbitacin B
CbL: Curcubitacin L
CbE: Curcubitacin E
L: Length
Ci: Consistency index
Ri: Retention
PC: Principal components

## Units

µg mL^−^^1^, mg g^−^^1^: Concentration
cm: Length
°Hue: Color
°Brix: Sugars
%: Quantity
